# Measuring the impact of manganese exposure on children’s neurodevelopment: advances and research gaps in biomarker-based approaches

**DOI:** 10.1186/s12940-016-0174-4

**Published:** 2016-08-30

**Authors:** Donna J. Coetzee, Patricia M. McGovern, Raghavendra Rao, Lisa J. Harnack, Michael K. Georgieff, Irina Stepanov

**Affiliations:** 1 Medical School, University of Minnesota, 420 Delaware Street SE, Minneapolis, MN 55455 USA; 2 Division of Environmental Health Sciences, School of Public Health, University of Minnesota, Mayo Building, MMC 807, 420 Delaware St. SE, Minneapolis, MN 55455 USA; 3 Neonatology Division, University of Minnesota, Mayo Mail Code 391, 420 Delaware St. SE, Minneapolis, MN 55455 USA; 4 School of Public Health, University of Minnesota, 300 West Bank Office Building 1300 S 2nd St, Minneapolis, MN 55454 USA; 5 Martin Lenz Harrison Land Grant Chair, Pediatrics and the Institute of Child Development, Center for Neurobehavioral Development, University of Minnesota, 717 Delaware Street SE, Ste. 333, Minneapolis, MN 55414 USA; 6 Division of Environmental Health Sciences, School of Public Health, University of Minnesota, 420 Delaware St SE, MMC 807, Minneapolis, MN 55454 USA

**Keywords:** Manganese, Exposure biomarkers, Exposure measurement, Children’s neurodevelopment

## Abstract

**Background:**

Children’s exposure to manganese (Mn) is a public health concern and consistent policy guidelines for safe levels of Mn exposure is lacking. The complexity of establishing exposure thresholds for Mn partially relates to its dual role as an essential micronutrient with low levels required for good health, but also as a neurotoxin at high levels. Questions exist about the age-related susceptibility to excess Mn, particularly for children, and how best to measure chronic exposures. To address this concern we conducted a systematic review of studies examining children’s exposure to Mn and neurodevelopmental outcomes focused on selection of biomarker-based and environmental measurements of Mn exposure to identify the scientific advances and research gaps.

**Methods:**

PubMed and EMBASE databases were searched through March 2016 for studies that were published in English, used a biomarker-based or environmental measurement of Mn exposure, and measured at least one neurological outcome for children aged 0–18 years. Ultimately, thirty-six papers from 13 countries were selected. Study designs were cross-sectional (24), prospective cohorts (9), and case control (3). Neurodevelopmental outcomes were first assessed for Mn exposure in infants (6 papers), toddlers or preschoolers (3 papers) and school-age children (27 papers).

**Results:**

Studies of school-aged children most frequently measured Intelligence Quotient (IQ) scores using Mn biomarkers of hair or blood. Higher hair concentrations of Mn were consistently associated with lower IQ scores while studies of blood biomarkers and IQ scores had inconsistent findings. Studies of infants and toddlers most frequently measured mental and psychomotor development; findings were inconsistent across biomarkers of Mn (hair, cord blood, tooth enamel, maternal or child blood and dentin).

Although few studies measured environmental sources of Mn, hair biomarkers were associated with Mn in drinking water and infant formula. Only one paper quantified the associations between environmental sources of Mn and blood concentrations.

**Conclusion:**

Hair-Mn was the more consistent and valid biomarker of Mn exposure in school-aged children. Accurate measurement of children’s exposure to Mn is crucial for addressing these knowledge gaps in future studies. However, research on biomarkers feasible for fetuses and infants is urgently needed given their unique vulnerability to excessive Mn.

## Background

Neurodevelopmental disabilities exact a significant toll on children. The global burden of attention deficit hyperactivity disorder (ADHD)/hyperkinetic disorder (HD) was estimated at 5.3 % in 2006 with acknowledgement of the limitations of pooled national estimates [1]. Recent prevalence estimates from the United States (U.S.) identify 4.9 million (8 %) children are learning disabled and another 5.9 million (9.5 %) have attention deficit disorder [2]. The number of U.S. children diagnosed with learning and behavioral problems has increased with time. From 1998–2000 through 2007–2009, the prevalence of ADHD among children aged 5–17 years grew from 6.9 % to 9.0 % [3]. Smaller, subclinical decrements in brain function are more common than diagnosed disorders and such conditions may decrease children’s academic success, disturb behavior, and diminish quality of life [4]. These conditions are associated with a growing list of potential neurotoxicants including manganese (Mn). As with most divalent metals (e.g., iron, lead and cadmium), excessive environmental exposure to Mn adversely affects the brain function in adult humans and pre-clinical (animal) models of maternal-fetal dyads. The health implications for fetuses and infants are a concern given the propensity for Mn accumulation in tissue is higher during development [5], and their rapidly developing brain may be at risk of injury at lower levels of Mn exposure, relative to older children and adults [6, 7]. However, the potential adverse effects of excessive levels of Mn on the infant brain are poorly understood. Manganese is an essential micronutrient that plays a critical role in normal growth and development, particularly for brain development [8]. Humans need Mn in their daily diet because it is required for normal amino acid, lipid, protein, and carbohydrate metabolism [6]. Mn deficiencies are considered rare because Mn is present in numerous commonly consumed food items such as seafood, nuts, spinach, and tea. However, overexposure to Mn is also detrimental to health. Accumulation of Mn in the brain results in neurotoxic effects. Neurons in their early developmental stage are especially sensitive to the neurotoxic effects of Mn [9]. Animal studies demonstrate that Mn uptake by the brain is higher in the pre-weaning period, relative to later ages. Exposure to excess Mn in the prenatal and postnatal periods leads to tissue Mn deposition in the striatum and hippocampus [5, 7], brain regions that are important for cognitive function. Increased startle, hyperactivity, and learning and memory deficits are the functional consequences of exposure to excess Mn during development in rats [7, 10]. Some of these effects are long-term and persist into adulthood, despite the cessation of exposure to excess Mn [11]. Mn neurotoxicity is greater with combined prenatal and postnatal exposures than with exposure limited to either prenatal or postnatal period, and is mediated by altered neurotransmission, neuronal apoptosis and mismigration, excitotoxicity and oxidative stress [5]. In addition, Mn may indirectly affect brain function by altering tissue homeostasis of other divalent metals that are important for normal neurodevelopment, such as iron, by altering the expression of transporters that are common to all divalent metals [12].

In adult humans, excess Mn may result in anxiety, learning and memory deficits, and motor impairment [13, 14]. Inhalation of Mn is a long-standing concern for workers in the ferromanganese, iron and steel mining, welding and battery assembly industries that contain extremely high levels of Mn (>1–5 milligrams Mn/meter,^3^ or mg Mn/m^3^) [6]. Community exposures to Mn also exist and include air contaminants from industrial activities [15], residential proximity to hazardous waste [16] or ingestion of water with naturally occurring Mn [17, 18]. Mn inhalation may bypass the biliary excretion mechanism and enter the brain through facilitated diffusion and active transport across the blood-brain barrier [19], or be passively transported from the olfactory bulb to the cerebral cortex [20], Mn has been considered to be less toxic when ingested than inhaled because adult humans regulate Mn absorption in the gastrointestinal tract and usually excrete excess Mn taken orally [21]. However, infants’ regulatory system is immature thus the risk of tissue Mn accumulation is greater for fetuses and infants [6, 22] raising questions about a World Health Organization’s (WHO) [23] decision to suspend guidelines addressing Mn concentrations in water [24].

The former WHO drinking water guideline of 400 micrograms/L for Mn was withdrawn in 2011 as unnecessary with an assertion that this health-based level was well above Mn concentrations normally found in drinking water [23]. However, Frisbie and colleagues report that over 50 countries have drinking water or potential drinking-water supplies that contain a Mn concentration greater than 400 micrograms /L and argue that protective policy guidance is needed [24]. The US Environmental Protection Agency (EPA) provides Health Advisory (HA) values for unregulated contaminants that may cause non-cancerous health effects. EPA has identified that a lifetime HA at 0.3 mg/L Mn in water is not expected to cause adverse neurological effects [25]. While age-specific exposure limits are not available, for infants younger than 6 months, the lifetime HA of 0.3 mg/L Mn in water is recommended for acute exposures of 10 days, given concerns for differences in Mn content in human milk and formula and the possibility of a higher absorption and lower excretion in young infants [25].

A rapidly growing body of literature reveals the complexity of the association between exposure to Mn and children’s adverse neurodevelopmental outcomes given a child’s age, developmental and nutritional status (e.g., hemoglobin levels). However, the levels, timing and duration of exposure at which these outcomes may occur, and the potential effect of various routes of exposure to Mn (e.g., drinking water, dietary practices and contaminated air or soil), are not well established. Furthermore, the mechanisms of Mn toxicity are poorly understood and are complicated by interactions with other toxic metals such as lead (Pb) [26–28] and arsenic (As) [16, 29, 30] and limited and inconsistent evidence of gender-specific neurological effects (generally greater effects in girls [31, 32], but also found in boys) [33]. Accurate measurement of children’s exposure to Mn is critical to address these knowledge gaps in future studies. Our paper examines the evidence for the association of Mn exposures to children’s neurodevelopmental outcomes, focused on the contribution of biomarkers and environmental measures for elucidating the exposure-outcome relationship.

## Methods

We identified studies using PubMed and EMBASE search engines in March of 2016. The searches were conducted by combining the results from a search on ‘manganese’ combined with the results from a strategy that used the concept of neurological outcomes including the following keywords: ‘neurobehavioral manifestations’ or ‘intelligence’ or ‘child behavior’ or ‘child development’ or ‘psychomotor performance’ or ‘neuropsychological tests’ or ‘psychomotor disorders’ or ‘cognition’ or ‘intelligence test’ or ‘intelligence quotient.’ The inclusion criteria were that the article was published in English and reported a study that measured both Mn exposure and a neurological outcome in humans aged 0–18 years. Any study that met the selection criteria, regardless of the publication date, was included in an initial phase of review. Measurements of Mn exposure varied including biomarkers and environmental sources; both types of exposure measurements were included. While various neurological outcomes were assessed, no limits were placed on the types of neurological outcomes examined.

## Results

The searches returned 132 unique references. Fifty-six papers were outside the scope of this review because they were published in languages other than English, were review articles or meeting abstracts, had animal subjects, or did not include both a measure of Mn exposure and a neurological outcome. Abstracts were reviewed for the remaining 76 articles; ultimately 36 papers met all selection criteria and were included in this paper (Table 1).

**Table 1:** Summary of study characteristics

Study Author Date of Publication	Country	Study design	Study Population	Sample size	Environ-mental Mn Measure	Biomarker Measure	Neurodevelopmental Outcome
Barlow et al. (1983) [62]	United Kingdom	Case control	Children ≤ 16 years	68 exposed (65 controls)	None measured	Hair	Diagnosis of hyperactivity by personal physicians, child psychiatrists and social workers
Collipp et al. (1983) [34]	Long Island, New York, US	Cross-sectional	Infants and children ≤ 4 years	70	Infant formula	Hair	No health outcome
		Case control	Learning disabled children and controls 7–10 years	16 learning disabled children; (44 controls)		Hair	Learning disability for 7-10 year olds (parent and teacher reports, child interview, and neurological exam)
Takser et al. (2003) [43]	Paris, France	Prospective	Mother-infant pairs followed until 6 years	247 mother- infant pairs, 100 after 6 years)	None measured	Hair, cord blood, placenta	Attention, nonverbal memory, hand skills, general psychomotor indices (Brunet-Lézine development quotient at 9 months), (McCarthy General Cognitive Index at 3 and 6 years)
Wasserman et al. (2006) [18]	Araihazar Bangladesh	Cross sectional	Children 9.5–10.5 years	142	Well water	Blood	IQ (Wechsler Intelligence Scale for Children, WISC-III)
Wright et al. (2006) [16]	Miami, OK, US	Cross sectional	Children 11–13 years	31	Not measured, but location coexisted with a Superfund site (Pb, Zn, Mn, Cd)	Hair	IQ (Wechsler Abbreviated Scale of Intelligence or WASI); Wide Range Assessment of Visual Motor Ability; receptive scales of Clinical Evaluation of Language Fundamentals; California Verbal Learning Test-Children; Behavioral Assessment System for Children; tests of story memory (Wide Range Assessment of Memory and Learning); Children’s Depression Inventory, and the Behavior Rating Inventory of Executive Functions
Bouchard et al. (2007) [17]	Québec, Canada	Cross sectional	Children 6–15 years	46	Well water	Hair	Hyperactivity, oppositional behavior, cognitive problems, inattention, (Revised Conners Teachers’ and Parents’ Rating Scales)
Ericson et al. (2007) [52]	United States	Prospective	NICHD Study of Early Child Care and Youth Development who shed a tooth	27	None measured	Tooth enamel	Behavioral disinhibition (Forbidden Toy Task), sustained attention (Mirsky Continuous Performance Test), impulsive error score (Children’s Stroop Test), and total, internalizing, externalizing and attention problems (Children Behavior Checklist)
Kim et al. (2009) [26]	Seoul, Seongnam, Ulsan, and Yeoncheon, South Korea	Cross sectional	Children 8–11 years	261	None measured	Blood	IQ including vocabulary, arithmetic, picture arrangement and block design (Korean Educational Development Institute-Wechsler Intelligence Scales)
Claus Henn et al. (2010) [44]	Mexico City, Mexico	Prospective	Children enrolled at or before birth and followed through age 3	448	None measured	Blood	Mental Development Index (MDI) and the Psychomotor Development Index (PDI) (Bayley Scales of Infant Development-II)
Riojas- Rodriguez et al. (2010) [31]	Hidalgo, Mexico	Cross sectional	Children 7–11 years	79 (93 controls)	None measured	Hair, blood	IQ (WISC-Revised)
Bouchard et al. (2011) [32]	Québec, Canada	Cross sectional	Children 6–13 years	362	Water, diet	Hair	IQ (WASI)
Hernández-Bonilla et al. (2011) [48]	Hidalgo, Mexico	Cross sectional	Children 7–11 years	100 exposed (95 controls)	Prior studies show airborne Mn levels (median 0.10 g/m^3^) exceed 2006 US EPA Reference Concentration (0.05 μg/m^3^)	Hair, blood	Motor function: grooved pegboard, finger tapping, and Santa Ana test
Khan et al. (2011) [29]	Araihazar Bangladesh	Cross sectional	Children 8–11 years	201	Water	Blood	Child behaviors including internalizing and externalizing subscales and a total score (TRF Achenbach System of Empirically Based Assessment)
Menezes-Filho et al. (2011) [35]	Salvador, Brazil	Cross sectional	Children 6–12 years	83	None measured	Hair, blood	IQ (WISC - III)
Parvez et al. (2011) [49]	Araihazar Bangladesh	Cross sectional	Children 8–11 years	303	Drinking water	Blood, toenails	Motor function (Bruininks-Oseretsky Test) including total score and subscales for coordination of hands and fingers and arms and hands, body coordination, strength and agility
Khan et al. (2012) [58]	Araihazar Bangladesh	Cross sectional	Children 8–11 years	840	Water	None measured	Academic achievement from nationwide exams in math and language
Wasserman et al. (2011) [30]	Araihazar Bangladesh	Cross sectional	Children ages 8–11 years	299	Well water	Blood	IQ, (WISC-IV) including verbal comprehension, perceptual reasoning, working memory, processing speed, and full scale scores
Claus Henn et al. (2012) [27]	Mexico City, Mexico	Prospective	Children enrolled prenatally; followed to 36 months	455	None measured	Blood	Bayley Scales of Infant Development-II (MDI and PDI)
Lucchini, Zoni, et al. (2012) [41]	Valamonica and Garda Lake, Italy	Cross sectional	Children 11–14 years	299	PM10, soil	Hair, blood, urine	IQ (WISC-III) including the overall, verbal and performance scores), and behavior (Conners-Wells’ Adolescent Self-Report Scale Long Form)
Lucchini, Guazzetti et al. (2012) [33]	Valamonica and Garda Lake, Italy	Cross sectional	Children 11–14 years	54 exposed (157 control)	PM10, soil, tap water, diet	Hair, Blood	Motor coordination (Luria Nebraska Battery) including hand dexterity, odor identification and tremor intensity
Bhang et al. (2013) [40]	South Korea	Cross-sectional	Children 8–11 years	1005	None measured	Blood	IQ (WASI), ADHD Diagnostic System, Stroop Color-Word Test, Children’s Color Trails Test, ADHD Rating Scale, Learning Disability Evaluation Scale, Child Behavioral Checklist and the Diagnostic Interview Schedule for Children-IV
Torres- Agustín et al. (2013) [54]	Hidalgo, Mexico	Cross sectional	Children 7–11 years	79 (95 control)	PM10, soil	Hair, Blood	Memory and learning (Children’s Auditory Verbal Learning Test) including learning curve and level, immediate and delayed recall, recognition accuracy and immediate memory span
Lin et al. (2013) [28]	Taipei, Taiwan	Prospective	Mother-Infant pairs in the Taiwan Birth Panel	230 (pairs)	None measured	Cord blood	Development (Comprehensive Developmental Inventory for Infants and Toddlers) global, cognitive, language, motor, gross motor, fine motor, social, self-help, and behavioral development.
Carvalho et al. (2014) [36]	Simões-Filho district, Bahia, Brazil	Cross sectional	Children 7–12 years	70	None measured; participants lived near a ferromanganese alloy plant	Hair	IQ (WISC-III), short-term, and working memory, sustained attention problem solving (Wisconsin Card Sorting Test, WCST-64) and sustained attention (TAVIS-III)
Menezes- Filho et al. (2014) [53]	Salvador, Bahia, Brazil	Cross sectional	Children 7–12 years	70	None measured, but airborne exposure from residential proximity to ferromanganese plant	Hair, blood	Internalizing and externalizing behaviors, and attention problems (Children’s Behavioral Checklist)
Oulhote et al. (2014) [47]	Quebec, Canada	Cross sectional	Children 6–13 years	375	Tap water, water consumption	Hair	Memory and learning (California Verbal Learning Test-Children’s Version), Connors Continuous Performance Test II, Version 5, Digit Span, Santa Ana Test, manual finger tapping
Rink et al. (2014) [46]	Montevideo, Uruguay	Cross sectional	14–45 months old	60	None measured	Hair	Bayley Scales of Infant Development-III, cognitive, language, fine and gross motor abilities
Yang, et al. (2014) [57]	Shangai, China	Prospective	Mother-infant pairs	933	None measured	Cord blood	Neonatal Behavioral Neurological Assessment
Chung et al. (2015) [42]	Seoul, Ulsan and Cheonan, South Korea	Prospective	Maternal - infant pairs recruited prenatally	232 mother- infant pairs assessed at 6 months postpartum and followed for 3 years	None reported	Maternal blood	Bayley Scales of Infant Development
do Nascimento et al. (2015) [37]	Rio Grande do Sul, Brazil	Cross sectional	Children 6–12 years	69	Tap water	Hair, blood	Nonverbal IQ (Raven’s Colored Progressive Matrices)
Gunier et al. (2015) [45]	Salinas Valley, California, US	Prospective	Children recruited from prenatal cohort; followed to 7 years	197 (prenatal) 193 (postnatal)	Residential proximity to agricultural use of Mn- containing fungicides and ‘take home’exposures	Teeth (pre- and postnatal dentin from incisors)	Cognitive abilities, fine and gross motor coordination (Bayley’s Scales of Infant Development)
Haynes et al. (2015) [15]	Marietta, Ohio, US	Cross sectional	Children 7–9 years	404	None reported; PM2.5 associated with residential proximity to a ferromanganese refinery	Hair, blood	IQ (WISC-IV), including perceptual reasoning, processing speed, working memory and verbal comprehension
Mora et al. (2015) [39]	Salinas Valley, California, US	Prospective	Children enrolled prenatally provided shed teeth starting at 7–9 years, followed to 10.5 years	248 (prenatal) 244 (postnatal)	Residential proximity to agricultural use of Mn- containing fungicides	Teeth (pre- and postnatal dentin from incisors)	Behavior including internalizing, externalizing and hyperactivity (Behavioral Assessment System for Children; Conners’ Attention Deficit Hyperactivity Disorder; Statistical Manual of Mental Disorders, DSM-IV), accuracy and impulse control (Connors’ Continuous Performance Test II, Version 5) Cognition and memory including IQ (WISC-IV), verbal comprehension, perceptual reasoning, working memory and processing speed and full-scale score; visuospatial and verbal memory (NEPSY II Memory for Designs) verbal learning and memory abilities (CAVLT-II) Motor including fine motor dexterity including finger tapping and pegboard at age 7, and subtests of the Luria Nebraska Motor Battery, at ages 9 and 10.5
Ode et al. (2015) [50]	Malmö, Sweden	Case control	Children born 1987 to 2000 diagnosed with ADHD 5–17 years; matched controls	166 (case-control pairs)	None measured	Cord serum	ADHD diagnosis (Diagnostic and Statistical Manual of Mental Disorders, DSMMD-III, IV)
Shin et al. (2015) [51]	Seoul, South Korea	Case control	Children, 6–16 years, ADHD cases referred post-diagnosis	40 cases (43 controls)	None measured	Hair	ADHD diagnosis DSMMD- IV, Kiddie-Schedule for Affective Disorders and Schizophrenia-Present and Lifetime Version and other tests
Sun et al. (2015) [38]	Jiangsu, China	Cross sectional	Children, 8–12 years with natural environmental lead exposure	446	Mean community Pb concentrations in surface soil: 27.7 mg/kg, ^-1^ and undetected levels in outdoor air (<0.0035 mg/ m^-3^	Blood	IQ (Combined Raven Test modified in China)

Thirty six studies were conducted in thirteen countries investigating populations from the U.S. (six papers), Bangladesh and Mexico (five papers each), Brazil and South Korea (four papers each), Canada (three papers), Italy and China (two papers each), and France, Sweden, Taiwan, the United Kingdom and Uruguay (one paper each). Study designs were primarily cross-sectional (*N* = 24), and less frequently, prospective cohorts (*N* = 9), and case control (*N* = 3), although Collipp et al. [34] augmented the primary cross-sectional study with a secondary case control study (which is not included in the count of study designs). Sample sizes ranged from 16 (cases only) to 1,588 with approximately 7,639 children in total (except for children classified as controls in the case-control studies). Ten studies enrolled newborns. The ages at which neurodevelopmental outcomes were *first* assessed in relation to Mn exposure included infants (6 papers), toddlers or preschoolers (3 papers) and school-age children (27 papers). Exposure was more frequently measured with biomarkers (33 papers) than environmental samples (13 papers), (Table 1).

### Neurodevelopmental outcomes

Studies examining the potential for the adverse impact of Mn on neurological outcomes most frequently assessed measures of IQ [15–18, 26, 30–32, 35–41]*,* infant and toddler development [27, 28, 42–46], motor skills [33, 39, 47–49], attention deficit and hyperactivity disorder [39–41, 50, 51]*,* attention [36, 43, 52, 53] memory [15, 16, 30, 31, 36, 39, 43, 54] and behavioral problems [16, 17, 29, 39, 40, 52, 53]*.*

IQ was most frequently studied among children ages 7–14 years [15, 16, 18, 26, 30–32, 35, 36, 38–41] with the Wechsler Intelligence Scale for Children (WISC), consistent with its design for children ages 6 to 16 years and 11 months, using both full-scale IQ (global intelligence) and specific (verbal or performance) scores [55]. Study findings varied across study designs. Lower full-scale IQ scores were associated with increased concentrations of Mn in six studies investigating IQ as the only neurodevelopmental outcome [15, 26, 30, 32, 35, 37] and in three additional studies evaluating several neurological outcomes [16, 31, 36]. However, six studies did not report a significant association between IQ and Mn including studies only measuring IQ [18, 31, 35, 38] and one measuring additional outcomes [41]. In contrast to the former studies, Mora et al. examined several neurological outcomes and reported a positive association between postnatal Mn concentrations and IQ only for boys [39].

Motor function was measured in children 7 to 14 years and measures included grooved pegboard (a manipulative dexterity test), finger tapping [33, 39, 46, 47], the Santa Ana test which assesses manual dexterity and motor coordination [47, 48], the Bruininks – Oseretsky test which evaluates gross and fine motor functioning [49], the Aiming Pursuit test of hand dexterity [33] and subtests of motor coordination from the Luria Nebraska Battery [33]. Findings varied by study design and measures of outcomes. Mora et al. found higher concentrations of prenatal and postnatal Mn was associated with improved motor outcomes, but only in boys [39]. Oulhote et al. reported a significant association between intake of water-Mn and poorer motor function [47]. Hernández-Bonilla et al. reported a subtle, negative association of Mn with specific areas of motor speed and coordination [48]. Lucchini et al. reported higher Mn levels associated with poorer motor coordination and hand dexterity, and increased tremor intensity [33]. In contrast to the preceding studies, Parvez et al. did not find associations between Mn and motor function [49].

Among toddlers and infants ages 1 to 42 months the Bayley Scales of Infant Development (BSID-II) [56] were most frequently used to measure mental and psychomotor development [27, 42, 44, 46, 47]. A significant, inverted U-shaped association between Mn and development scores was reported in two studies. Chung et al. reported a dose-response relationship with both lower and higher concentrations of Mn associated with poorer Mental Development Index (MDI) and Psychomotor Development Index (PDI) scores in 6 month old infants [42]. Claus Henn et al. found an association between concurrent MDI scores at 12 (but not 24) months of age, but no association for PDI scores at either time period [44]. Two additional studies reported significant interactions of Mn and development scores by sex. A significant interaction of postnatal Mn exposures and poorer 6 month MDI and PDI scores and sex was reported by Gunier et al.; a significant inverse, linear relationship was seen only for girls [45]. A significant, positive interaction between postnatal Mn and sex was also seen at 24 months, but only for boys who had better MDI scores [45]. Rink et al. also reported a positive association between Mn and MDI scores only in boys, on average 29 months of age [46].

### Biomarkers

Studies generally used biomarkers of children’s hair or blood to assess Mn, but a few investigators measured fetal cord blood or serum, maternal blood or children’s enamel or dentin from shed teeth; one study measured urine, (Tables 2, 3 and 4). Hair-Mn was the biomarker most consistently associated with a range of neurodevelopmental deficits. Higher levels of hair-Mn in school-aged children were significantly, and inversely associated with IQ scores [15, 16, 31, 32, 35–37], learning [16, 54], memory [16, 36, 54], perceptual reasoning [15] and positively related with greater hyperactive and oppositional behavior [17, 34].

**Table 2:** Summary of results from studies examining manganese concentrations in hair (Hair-Mn or H-Mn)

Study	Children’s Ages and Mean Mn Level (μg/g), (SD)	Association with Environmental Mn	Association with Neurodevelopment	Other metals’ mean concentrations in hair (μg/g), (SD)
Barlow et al. (1983) [62]	<16 years Hyperactive: 0.84 (0.64) Control: 0.68 (0.45)	None measured	Hyperactivity was more prevalent in hyperactive children (mean age: 7.6 years) but at lower levels of statistical significance (90 % confidence) using bivariate analyses.	Lower levels of zinc (Zn) were associated with hyperactive children: 83.4 (32.3) compared to controls: 99.1 (54.3), 95 % confidence using bivariate analysis. Other metals were nonsignificant in association with the outcome including: (Cadmium (Cd), Copper (Cu), Iron (Fe), Lead (Pb), and Magnesium (Mg).
Collipp et al. (1983) [34]	*Ages 7–10 years* Learning- disabled:0.43 Control: 0.27	None measured	Significantly higher hair-Mn levels from learning disabled children, 7–10 years old, compared to children without the condition.	Not applicable
	*Age 4-months* Breastfed: 0.33 Formula fed: 0.685	Significantly greater hair-Mn in formula-fed infants.	None applicable	Not applicable
Takser et al. (2003) [43]	Newborns to 6 years 0.75^1^	None measured	No association was found between hair-Mn post-childbirth and general psychomotor developmental indices at 9 months and a general cognitive index at 3 and 6 years in models adjusted for maternal age and education, smoking, labor duration, children’s sex and cord blood lead levels and other confounders.	Not applicable
Wright et al. (2006) [16]	11–13 years 0.47	None measured	Lower full-scale IQ, verbal learning and memory scores were associated with higher concentrations of hair-Mn from children, on average 12.6 years old, in analyses adjusted for maternal education, child sex and concentrations of lead PbH.	Higher arsenic (As) levels, particularly in combination with higher Mn levels, associated with lower IQ, verbal learning, and memory scores. No associations found with Cd levels.
Bouchard et al. (2007) [17]	6–15 years 5.1 (4.3)	Greater MnH concentrations from children who drank well water with higher Mn-water.	Greater hyperactive and oppositional classroom behavior was associated with higher hair-Mn from children, on average, 11 years old, in analyses adjusted for age, sex and income. No interaction between hair-Mn and child sex.	Not applicable
Hernández-Bonilla et al. (2011) [48]	7–11 years 12 (exposed) 0.57 (nonexposed)	Respiratory Mn exposures were associated with residential proximity to Mn mines, but specific measures were not reported.	Hair-Mn was not associated with neuromotor outcomes (grooved pegboard, finger tapping repetition and Santa Anna test in children, on average, 9 years old in analyses adjusted for Pb in blood, hemoglobin, sex, age and maternal education.	Not applicable
Menezes- Filho et al. (2011) [35]	6–12 years 5.83^1^ (11.5) Hair-Mn levels were 6 times higher than those in the general Brazilian population (mean 0.47 μg/g, range 0.89–2.15 μg/g)	None measured, but Mn exposures were from residential proximity to Mn alloy production plant.	Lower full scale and verbal IQ scores in children, on average, 8.8 years old, in analyses adjusted for maternal education and nutritional status. A ten-fold increase of hair-Mn was associated with a 6.7 - point loss in Verbal IQ score.	Children with iron deficiency had higher hair-Mn (15.94 ± 19.68 μg/g; *p* = 0.06) compared to those with FeS in normal range (8.69 ± 8.23 Mn/g).
Riojas- Rodriguez et al. (2010) [31]	7–11 years Exposed: 12.13 Unexposed: 0.57	Median airborne concentration of Mn in PM10 of exposed (0.13 μg/m^3^) versus unexposed (0.02 μg/m^3^) communities, but personal exposures were not reported.	Lower full scale, verbal and performance IQ scores in children, on average, 9 years old, in analyses adjusted for blood-Pb, hemoglobin, age, sex and nutritional status. Sex significantly modified the association with the strongest inverse association in young girls. There was little evidence of an association in boys.	Not applicable
Bouchard et al. (2011) [32]	6–13 years Median: 0.7	Hair-Mn levels were associated with higher Mn in water (Water-Mn) (mean: 98 μg/L, GM:^1^20 μg/L), but not in diet.	Lower full-scale IQ scores were associated with increased hair-Mn concentrations in children, on average, 9 years old, in analyses adjusted for maternal intelligence and education, income, sex and age of children, Fe concentrations in water and other confounders. A 10-fold increase in water-Mn was associated with a decrease of 2.4 IQ points (95 % CI:-3.9 to -0.9, *p* < 0.01) adjusting for maternal intelligence and other confounders. Sex stratification showed a slightly higher impact of hair-Mn for girls’ full-scale IQ, but the interaction term was nonsignificant. Water-Mn was more strongly associated with performance than verbal IQ.	Not applicable
Lucchini, et al. (2012) [33]	11–14 years 0.16 Median	Significant differences for Mn concentrations in soil (soil-Mn) and air (air-Mn) by proximity to industrial sites with historical Mn emissions, but not for tap water, diet or hair. Impairment of motor coordination, hand dexterity and odor identification was associated with median concentrations of soil-Mn in exposed (897 ppm) versus reference (409 ppm) communities.	Tremor intensity in dominant hand was positively associated with hair-Mn in children, on average, 12.9 years old, in analyses adjusted for age, gender, SES, family size, parity order, parents’ education, smoking habits and soil concentrations of Pb and other metals. Boys had increased tremor intensity relative to girls.	Not applicable
Lucchini, et al. (2012) [41]	11–14 years 0.17	No association between concentrations of hair-Mn with soil- or air-Mn	No association between hair-Mn concentrations with full-scale, verbal or performance IQ, or behavioral and attention deficit hyperactivity scores for children, on average, 12.9 years old, with Mn exposure modeled as a main effect or an interactive term with blood-Pb in analyses adjusted for age, gender, family size, SES, area of residence, hemoglobin, ferritin and confounders.	
Torres- Agustín et al. (2013) [54]	7–11 years Exposed: 14.2 Unexposed: 0.73	Greater hair-Mn concentrations in children in exposed group. Mn concentrations in outdoor air from Mn mining and ranged from the median: 0.08, μg/m^3^ in the exposed location compared to the median: 0.02, μg/m^3^ in the control location.	Lower long-term memory and learning scores were associated with increased hair-Mn in children, on average, 9 years old, in analyses adjusted for children’s sex, blood-Pb, age, hemoglobin and maternal education. The negative association was stronger for girls.	Not applicable
Rink et al. (2014) [46]	14–45 months 0.98 (0.74)	None measured	Lower scores in cognitive and expressive language tests in children, on average, 28.8 months old, but only in unadjusted models. Boys had a significantly positive association between hair-Mn concentrations and receptive languages scores in analyses adjusted for hair-Pb concentrations, child hemoglobin and age, paternal education, maternal IQ, SES, and other confounders.	Not applicable
Carvalho et al. (2014) [36]	7–12 years 14.6 (11.8)	None were measured, but Mn exposure was related to residential location and air emissions from an iron-Mn alloy plant.	Lower full-scale IQ, and lower scores on Vocabulary, Block Design, and Digit Span tests were associated with increased hair-Mn for children, on average, 9.4 years old, in analyses adjusted for maternal education and children’s age. Each 1 μg/g increase in hair-Mn was associated with a decrease of approximately 1 full-scale IQ point and lower test scores for executive function, strategic visual formation and verbal working memory. No significant sex differences for hair-Mn concentrations.	
Menezes- Filho et al. (2014) [53]	7–12 years Boys: 15.3 (9.9) Girls: 13.9 (13.4)	None were measured, but Mn exposure was related to residential location and air emissions from an iron-Mn alloy plant.	Externalizing behaviors and attention problems on the Child Behavior Checklist (CBC) for girls was significantly associated with higher hair-Mn. No significant association was found between CBC scores for boys in sex-stratified models adjusted for age (with boys) or maternal IQ (with girls).	
Oulhote et al. (2014) [47]	6–13 years Boys: 0.75 Girls: 0.80	Greater water-Mn (mean: 99 μg/L; GM: 20 μg/L) & hair.	Mn exposure was associated with significant decrements in memory (hair and water) and attention (hair), and motor function (water) adjusted for maternal education and nonverbal intelligence, tobacco consumption, child sex, age and other confounders. Estimates of associations by sex were similar.	
do Nascimento et al. (2015) [37]	6–12 years Rural: 2.07 (2.6) Urban: 0.45 (0.2)	Greater Mn in drinking water (mean: 20 μg/L) rural sites and (mean: 1.0 μg/L) urban sites) associated with greater Mn levels in hair.	Lower (nonverbal) IQ scores were associated with hair-Mn and water-Mn concentrations for children, on average, 8.5 years old using models adjusted for age, gender and parental education.	Additional, similarly specified models were tested for the association of Pb, Cr, As, Hg, and Fe in hair on cognitive outcomes. Only hair-Fe showed a significant and inverse association with outcomes.
Haynes et al. (2015) [15]	7–9 years 0.42^1^ (0.002)	Air-Mn associated with home proximity to ferromanganese refinery.	Lower full-scale IQ and perceptual reasoning scores were associated with hair-Mn for children, 7–9 years old in analyses adjusting for blood-Pb, blood-Mn, serum creatinine, community of residence, child sex, parents’ IQ, education and parenting confidence. A U-shaped association was observed as children with hair-Mn concentrations > 747 μg/g had significantly lower IQ than children with hair-Mn concentrations between 207.2–747 μg/g (ß -3.66, 95 % CI: -6.9, -0.43). Children with hair-Mn levels < 207 had lower, but nonsignificant associations with full scale IQ than those with concentrations between 207.2–747 μg/g.	Not applicable
Shin et al. (2015) [51]	6–16 years Case: 0.31 (0.46) Control: 0.22 (0.10)	None measured	No association between hair-Mn and ADHD was found in children on, average, 9.7 years old when analysis was adjusted for confounders of age, sex and full-scale IQ.	Not applicable

^1^ Geometric Mean used

**Table 3:** Summary of results from studies examining manganese concentrations in blood (blood-Mn)

Study	Mean Mn Level (μg/L),(SD)	Association with Environmental Mn	Association with Neurodevelopment	Association of other metals’ mean (SD) concentrations in blood, with the outcome
Wasserman et al. (2006) [18]	10 year olds 12.8 (3.2)	No association was found with water-Mn (Mean: 795 μg/L).	No association was found between blood-Mn concentrations and overall, verbal and performance IQ scores in adjusted analyses, but water-Mn was associated with lower full-scale, performance and verbal IQ raw scores in a dose-dependent fashion.	Blood-Mn was not significantly correlated with blood-Pb or blood-As. When all three blood metals were included in analyses only mean blood-Pb concentrations,12 μg/dL (3.7) were associated with IQ scores.
Kim et al. (2009) [26]	8–11 years olds 14.3 (3.8)	None measured	Lower overall and verbal (but not performance) IQ scores were associated with blood-Mn in analyses adjusted for maternal age, parental education and smoking, SES, child gender and age and other confounders.	Blood-Pb concentrations of 1.73 μg/dL (0.8) were associated with IQ scores in adjusted analyses with evidence of an additive interaction with blood-Mn. Effect modification was suggested as IQ scores of children with blood-Mn > 14 μg/L were significantly associated with blood-Pb whereas scores for children with blood-Mn < 14 μg/L were not.
Claus Henn et al. (2010) [44]	12 month: 24.3 (4.5) 24 month: 21.1 (6.2)	None measured	Blood-Mn had an inverse, U-shaped association with a concurrent measure of the Mental Development Index (MDI) scores at 12 months of age. Declines of 3.4 and 2.8 MDI points for the lowest and highest quintiles of blood-Mn relative to the middle three quintiles, correspond to declines of 0.37 and 0.31 SD units in the MDI. This association declined by 24 months and was nonsignificant in adjusted analyses including blood-Pb, sex, maternal IQ and education, hemoglobin and gestational age. No association was found with the PDI score.	Blood-Pb (cord, 12 and 24 month) concentrations were positively associated with 24 month blood-Mn concentrations. Indices of iron status (hemoglobin, ferritin) were inversely associated with Mn at 12 and 24 months of age.
Riojas-Rodriguez et al.(2010) [31]	7–11 year olds 9.7^a^ (Exposed) .2 (Control)	24-h median Mn in PM10 for the exposed (0.13 μg/m^3^) and control (0.02 μg/m^3^) communities	Exposed children showed nonsignificant, inverse associations of blood-Mn with lower full scale, verbal and performance IQ scores compared to controls. Analyses were adjusted for age, sex, hemoglobin, maternal education, blood-Pb. Differences by sex were nonsignificant.	Blood-Pb was higher in control (7.96 μg/dL) versus Mn-exposed (3.37 μg/dL) children and was correlated with blood-Mn (*r* - 0.24) in the population. It was not significantly associated with IQ outcomes.
Hernández-Bonilla et al. (2011) [48]	7–11 year olds 9.5 (Exposed) 8.0 (Control)	Prior studies show airborne Mn levels (median 0.10 μg/m^3^) exceed EPA 1999 Reference Concentrations (0.05 μg/m^3^).	Blood-Mn was inversely associated with poorer finger tapping in analyses adjusted for age, sex, maternal education, hemoglobin and blood-Pb. Other motor function measures (grooved pegboard and Santa Anna test scores) were not significantly associated with blood-Mn. Sex differences for blood-Mn were nonsignificant.	Blood-Pb concentrations were higher in the Mn control (median: 8 μg/dL) versus the Mn exposed (median: 3.3 μg/dL) children. The associations with the outcomes were not reported.
Kahn et al. (2011) [29]	8–11 year olds 15.1 (3.9)	Non-significant association of blood-Mn with water-Mn (mean: 900 μg/L).	No association was found between blood-Mn and externalizing (attention problems and aggression) and internalizing (anxiety) behaviors and a total behavioral score in analyses adjusted for water-As, water-Mn, urinary creatin-adjusted As and blood-As, sex, maternal education and other variables.	There was no statistical association between biomarkers of As (blood or urine) with blood-Mn.
Menzes-Filho et al. (2011) [35]	6–12 year olds 8.2 (3.6)	None measured; Mn exposure was due to home proximity to Mn alloy production.	Blood-Mn concentrations were not associated with IQ scores in analyses adjusted for blood-Pb or low serum iron levels.	Blood-Pb was above 2 μg/dL for 51 % (*n* = 36) and there was no association with blood-Mn or serum-Fe (mean: 55.6 μg/dL).
Parvez et al. (2011) [49]	8–11 year olds 17.7 (3.7)	Water-Mn (mean: 725.5 μg/L). Children with higher water Mn (>500 μg/L) did not have higher levels of blood-Mn (14.5 vs. 15.0 μg/L; *p* < 0.05).	No significant associations were found between blood-Mn and motor function measures (fine manual control, manual and body coordination, strength and agility).	Blood-Mn correlated slightly with blood-As (mean: 4.8 μg/L; SD: 3.2; r = 0.12; *p* = 0.02) and moderately with blood-Se (mean: 104.9 μg/L; SD:17.2; r = -0.33, *p* < 0.0001). There was a significant, inverse association between As exposure measures (blood, water, urinary and nails) and overall motor function, and a significant association between blood-Se and manual coordination in adjusted analyses. No significant association was found between blood-Pb and motor function.
Wasserman et al. (2011) [30]	8–11 year olds 14.78 (3.7)	Water-Mn (mean: 725.54) and blood-Mn did not vary predictably across groups with high and low levels of water-Mn.	Higher blood-Mn was associated with lower perceptual reasoning and working memory scores in analyses adjusted for maternal intelligence and age, children’s time in school, plasma ferritin, blood-As and other variables. Significant associations were not found for full scale IQ, verbal comprehension or processing speed scores.	Increased concentrations of blood-As (mean: 4.81 μg/L; SD: 3.22) were significantly associated with lower verbal comprehension in adjusted analyses. However, Mn by As interactions were not significant in adjusted models predicting IQ.
Claus Henn et al. (2012) [27]	12 months: 24.7 (5.9) 24 months: 21.5 (7.4)	None measured	A synergistic interaction between lead and Mn for mental and psychomotor development scores was found at 12 (but not 24) months; greater lead toxicity with higher Mn levels in analyses adjusted for sex, hemoglobin, gestational age, maternal education and IQ. There were no significant sex differences in blood-Mn.	Concentrations of blood-Pb at 12 (mean:5.1 μg/dL; SD: 2.6) and 24 months (mean: 4.8 μg/dL; SD: 2.5).
Lucchini et al. (2012) [33]	11–14 year olds 11.11 μg/dL	Mn was measured in air PM10 airborne particles (median: 31.4 ng/m^3^ vs. 24.7 ng/m^3^) and soil (median:897 ppm vs. 409 ng/m^3^) in impacted compared to control areas, and water (below LD at 1 μg/L) and diet (median 2.66 mg/day) with no differences by locations. Soil-Mn was significantly, inversely associated with performance on the olfactory test.	Tremor intensity, dominant hand, was significantly and positively associated with blood-Mn in adjusted models (including parental smoking and alcohol use, and Mn in soil, air and hair). Sex differences were found with boys having lower increased tremor intensity.	Blood-Pb concentrations in the Mn exposed (mean: 1.72 μg/dL) and control (mean: 1.6 μg/dL) communities were very low.
Lucchini et al. (2012) [41]	11–14 year olds 11.11 μg/dL	Mn was measured in soil (median: 529.12 ppm), air: (median: 29.37 μg/m^3^), water, and diet	Mn was not associated with IQ (full scale, verbal and performance) or behavioral (hyperactivity, attention deficit) scores in adjusted analyses.	Blood-Pb concentrations averaged 1.71 μg/dL and were adversely associated with cognitive measures in adjusted analyses declining about 2.4 IQ points with a two-fold increase of blood-Pb. A bench-mark level of blood-Pb was associated with loss of 1 IQ point at 0.19 μg/dL and a lower 95%CI of 0.11 μg/dL. No interaction of Pb and Mn was observed.
Torres-Agustín et al. (2013) [54]	7–11 year olds Exposed: 9.5^b^ Unexposed: 8.0	Air sampling (PM10) conducted and Mn concentrations in outdoor air from Mn mining significantly higher for exposed (Outdoor median: 0.08 mg/m^3)^ versus comparison group (Outdoor median: 0.08 mg/m^3^) Significantly greater blood-Mn concentrations in exposed than comparison children.	No significant associations between blood-Mn and verbal learning or memory in adjusted analyses.	Blood-Pb concentrations were significantly higher in the comparison group (8.0 μg/dL) than the Mn exposed group (3.3 μg/dL) and included in multivariate models of Mn exposure.
Bhang et al. (2013) [40]	8–11 year olds 14.42 (4.1)	None measured	Excess blood-Mn was associated with lower scores in thinking, reading, calculation, and learning scores and higher cognitive inhibition test scores in analyses adjusted for maternal and child age and IQ, child sex, and age, cotinine, blood-Pb and other variables. Lower blood-Mn was associated with lower cognitive inhibition scores.	Analyses were adjusted for blood-Pb and cotinine.
Chung et al. (2015) [42]	Maternal, pre- delivery, 30.1 ± 3.5 years; 22.5 (6.5)	Not measured	Inverted U-shaped dose- response curve with lower psychomotor development scores in infants at 6 months with both low and high levels of Mn. Adjusted mean PDI (but not MDI) scores differed significantly across Mn concentration groups. No differences in effects by sex were observed.	None reported.
do Nascimento et al. (2015) [37]	6–12 year olds Rural: 16.0 (4.2) Urban: 19.0 (4.3)	Water-Mn concentrations differed significantly between rural (mean: 0.20 μg/L) and urban (mean:1.0 μg/L) children; associations with blood-Mn were not reported.	No significant associations found for blood-Mn and nonverbal IQ in analyses adjusting for age, parents education and child sex.	No associations were found between metals in blood and serum (Pb, Cr, As, Hg and Fe) and nonverbal IQ.
Haynes et al. (2015) [15]	7–9 year olds 9.67 (1.27)^c^ [2]	Mn exposure resulted from residential proximity to ferromanganese refinery although measurements relative to the blood-Mn and the cognitive outcomes were not reported.	Blood-Mn was significantly associated with lower full scale IQ, perceptual reasoning, lower processing speed scores in analyses adjusted for hair-Mn, serum cotinine, blood-Pb, and community residence. Full scale IQ scores among children in the highest quartile of blood-Mn (>11.2 μg/L) were significantly lower than scores in children with blood-Mn between 8.2 μg/L to 11.2 μg/L (-3.51 points; 95 % CI:-6.64, -0.38). Children with the lowest quartile of blood-Mn (<8.2 μg/L) also had lower full scale IQ scores than children in the reference group although findings were nonsignificant (-2.14 points; 95%CI: -5.37, 1.09). The perceptual reasoning and processing speed scores had the strongest negative associations with blood-Mn.	Correlations between biomarkers found statistically significant included: blood-Mn and serum ferratin (mean: 34.4 ng/mL; r - 0.19, *p* < 0.01), blood-Mn and blood-Pb (mean: 0.82 μg/dL; r - 0.13, *p* = 0.02), and serum cotinine (0.08 μg/L) and blood-Pb (r = 0.34, *p* < 0.0001). Blood-Pb was significantly associated with processing speed, but not full scale IQ or other subscales. Cotinine was significantly associated with full scale IQ, perceptual reasoning, working memory and verbal comprehension.
Sun et al. (2015) [38]	8–12 year olds 16.2 μg/L	Not measured	Blood-Mn was not significantly associated with IQ, but it was associated with urinary retinol binding protein (RBP) which was associated with blood-Mn.	Blood-Pb (GM: 33.7 μg/L) was significantly, inversely associated with IQ.

^a^ Geometric Means are given for exposed and control groups

^b^ Median values for BMn

^c^ Geometric Mean (GM) and Standard Deviation (GSD)

**Table 4:** Summary of results from studies examining manganese in teeth

Study Author and Publication Date	Sample and Mean Mn Level (μg/L), (SD)	Association with Environmental Mn	Association with Neurodevelopment	Association with Metals
Ericson et al. (2007) [52]	Children from a maternal prenatal cohort that provided shed molars at 11–13 years. Mn concentrations from teeth enamel were measured but values not reported.	None measured	Prenatal Mn levels, representing exposures from the 20^th^ gestational week were positively associated with behavioral outcomes: higher levels of disinhibition (36 months), impulsivity (4.5 years), externalizing and internalizing problems (1^st^ and 3^rd^ grades) and disruptive behaviors (3^rd^ grade). No differences on standardized tests of cognitive ability or achievement. Analyses were adjusted for mothers’ education, family income and child ethnicity. Postnatal Mn levels, representing exposures from gestational weeks 62–64, only correlated with teachers’ reports of externalizing behaviors (1^st^ and 3^rd^ grades).	No association between pre- and postnatal Mn (r = 0.13, NS [1]), Mn and Pb (prenatal r = 0.09, NS; postnatal r = -.08, NS). A significant association was seen with prenatal Mn and Fe (r = 0.74, *p* < 0.001) but not postnatal Mn (r = - .06, NS).
Gunier et al. (2015) [45]	Children from a maternal prenatal cohort provided shed teeth starting at age 7. Mn from dentin of deciduous teeth [2] Prenatal: 0.51 (0.19) Postnatal: 0.20 (0.23).	Not reported, but related to residential proximity or use of agricultural fungicides with Mn.	Prenatal Mn levels were not associated with MDI or PDI at 6, 12 or 24 months and no interactions by sex. A two-fold increase in postnatal dentin- Mn levels was associated with small, significant decreases in MDI at 6 and 12 months (but NS at 24 months). Postnatal dentin-Mn levels were inversely related (but NS) with PDI scores at 6 months, but not 12 or 24 months. Effect modification by sex was reported with significant interactions between prenatal Mn and maternal hemoglobin (HGB) in girls at 6 months.	Girls whose mothers had lower prenatal hemoglobin (HGB, <11.6 g/dL) had a decrease of 10.5 points (95%CI: -16.2, -4.8; *n* = 38) on the MDI and 11.6 points (95%CI: 19.3, -3.9) on the PDI per two-fold increase in prenatal Mn at 6 months. No interactions with blood-Mn and blood-Pb observed or any relationships with neurodevelopment at 24 months.
Mora et al. (2015) [39]	Children from two integrated prenatal cohort samples provided teeth at 7–9 years. Mn from dentin of deciduous teeth: Prenatal: 0.46 (1.48); Postnatal: 0.14 (2.47)	None reported, but exposure related to agricultural exposures to Mn-containing fungicides.	Behavior: No significant associations for prenatal Mn and behavioral outcomes in children ages 7, 9 or 10.5 years. Higher postnatal Mn was significantly associated with maternal reports of hyperactive, internalizing and externalizing behaviors for children aged 7 years, but not at older ages. Cognition: Neither prenatal nor postnatal Mn was consistently and significantly associated with cognitive outcomes. A sex effect was shown only for boys with a positive, significant relationship between postnatal Mn and cognitive scores (full scale, verbal comprehension, and perceptual reasoning IQ) at ages 7 and 10.5 years, and working memory IQ at 7 years. Memory: Higher prenatal dentin Mn levels associated with significantly better memory scores for children ages 9 and 10.5 and in sex stratified analyses. Postnatal Mn levels were not associated with memory scores in analyses of all children. Sex-stratified analyses revealed higher Mn significantly associated with better memory scores at 9 and 10. 5 years in boys. Motor function: No consistent, significant associations of prenatal Mn with motor function for all children. Sex-stratified analyses showed higher dentin Mn levels significantly associated with better motor function only in boys (finger tap Z-score at 7 years), Luria-Nebraska Motor Scale at 10.5 years). Postnatal Mn levels showed no consistent, significant associations for all children, but sex effects show higher dentin Mn levels associated with significantly better motor function scores only in boys at 7 years.	Higher prenatal Mn levels were associated (NS) with poorer visual spatial memory outcomes at 9 years and poorer cognitive scores at 7 and 10. 5 years in children with higher Pb levels (≥0.8 μg/dL).

^a^ NS refers to a statistical association that is not significant

^b^ Geometric Means and Standard Deviations

IQ was the most frequently identified neurodevelopmental deficit associated with hair-Mn. Lower IQ scores were associated with increased concentrations of hair-Mn in four studies investigating IQ as the only neurocognitive outcome [15, 32, 35, 37], but IQ was also determined to be the only significant association with hair-Mn in a fifth study which measured several neurological outcomes [31]. Only one study found no significant association between IQ and Mn concentrations in hair [41]. In this study the mean Mn concentration in the hair was low, perhaps because Mn exposure in this study was from historical ferroalloy emissions.

Estimates of the effect size of hair-Mn on the average, full scale IQ scores of children, (mean age 9 years), were reported by Bouchard and colleagues to decline slightly (from 106 to 104) with hair-Mn values less than 1.5 micrograms/g, but significantly so for IQ scores of 101 with mean hair-Mn values of 3.2 micrograms/g, suggestive of biological significance [32]. Evidence of a U-shaped relationship with both high and low concentrations of hair-Mn associated with lower full scale IQ scores in children, on average, 8 years old were reported by Haynes et al. [15], suggestive of Mn as both a neurotoxicant and a micronutrient. Study findings revealed a significant, negative association between the highest quartile versus middle two quartiles of hair-Mn (ß -3.66; 95 % CI: -6.9, -0.43) and full scale IQ [15].

Blood-Mn levels were associated with a neurodevelopmental outcome in nine of sixteen papers reviewed. Most studies reporting associations between blood-Mn and neurological outcomes measured several outcomes. However, seven studies only examined IQ as the primary outcome and the findings were inconsistent. Four investigations did not find an association between IQ and blood-Mn [18, 31, 35, 38], but studies by Haynes et al. [15], Kim et al. [26], Wasserman et al. [30], showed a significant, inverse association between blood-Mn and IQ scores for children, on average, 8–9 years of age with mean concentrations of blood-Mn at 9.7 micrograms/L, 14.3 micrograms/L and 14.8 micrograms/L, respectively. Evidence of an inverse, U-shaped association between low and high levels of blood-Mn and low IQ scores was seen in three studies with children [15, 42, 44], two of which used the same outcome measure. Claus Henn et al. reported a significant association between concurrent MDI scores and blood- Mn in 12 month old infants comparing the middle three Mn quintiles with the lowest Mn quintile (ß -3.3, 95%CI: -6.0, -0.7) and the highest Mn quintile (ß -2.8, 95%CI: -5.5, -0.2) [44]. Chung and colleagues also reported a significant, inverse U-shaped association between maternal blood-Mn with infant PDI scores at 6 months. Increasing maternal blood-Mn levels up to 24–28 micrograms/L were positively associated with PDI scores while higher blood-Mn concentrations were associated with decreased PDI scores suggesting adverse effects of both low (<20 micrograms/L) and (high ≥ 30 micrograms/L) maternal blood-Mn levels [42].

Evidence for the usefulness of other Mn biomarkers included three papers that reported significant associations between Mn in cord blood or serum and early life neurodevelopment indicative of the importance of prenatal Mn exposure. Takser et al. reported an inverse association between cord blood-Mn at birth (Geometric Mean: 38.5 micrograms/L) and attention and non-verbal memory in three year olds and a significant, negative association with hand skills, significantly poorer scores in boys [43]. Lin et al. found cord blood-Mn (mean 50.7 micrograms/L; SD: 16.7 micrograms/L) and blood-lead (13.0 micrograms/L; SD: 7.51 micrograms/L) levels above the 75^th^ percentile had a significant association with overall (ß -7.03; SE = 2.56; *p* = 0.009), cognitive (ß -8.19, SE = 3.17; *p* = 0.012) and language scores (ß -6.81, SE = 2.73, *p* = 0.013) [28]. Yang et al. found that a high cord serum-Mn (≥75^th^ percentile, median: 4.0 micrograms/L) was associated with significantly lower scores on a Neonatal Behavioral Neurological Assessment (NBBA) at 3 days of age [57]. An interactive, protective effect was seen with prenatal selenium (Se); as the Mn/Se ratio increased, NBNA scores decreased while high levels of Se had a protective effect in the high Mn group (Mn ≥ 9.1 micrograms/L; Se ≥ 63.1 micrograms/L).

Teeth-Mn levels were analyzed in three studies suggestive of their potential value as biomarkers of early life exposures providing insight on the timing of Mn exposure and developmental windows of susceptibility. Ericson et al. measured tooth enamel in shed molars and found significant associations between Mn levels in enamel formed during the first 20 weeks of gestation and increased childhood behavioral inhibition at 36 months [52]. Studies from the Center for the Health Assessment of Mothers and Children of Salinas (CHAMCOS) birth cohort provided findings on the timing of early life Mn exposures. Gunier et al. reported small decreases in mental and motor development among 6 month old infants in association with prenatal dentin-Mn concentrations, but only for girls whose mothers had lower hemoglobin levels [45]. Additionally, a two-fold increase of postnatal dentin-Mn, reflecting exposures from birth to 2.5 months, was associated with a small, but significant decrease for infants’ mental development scores at 6 and 12 months. A significant interaction between postnatal dentin-Mn concentrations and sex for MDI (-1.5 points; 95 % CI: -2.4, -0.6) and PDI (-1.8 points; 95 % CI: -3.3, -0.3) scores at 6 months was reported, but only for girls; it was no longer evident by 24 months. Mora et al. reported increased Mn levels in pre-and postnatal dentin adversely associated with behavior problems in school aged children [39]. In contrast, the authors also reported positive effects of pre- and postnatal dentin-Mn specific to boys including better cognition, memory and motor function.

### Environmental sources of manganese

Levels of Mn in environmental sources were less frequently quantified than biomarkers of Mn. Collipp et al. found higher levels of hair-Mn in infants fed formula relative to breastfed infants [34]. Since the study’s publication, levels of Mn in infant formula have declined. This study was one of the first published papers to show an association between ingestion of dietary Mn (formula) and hair biomarkers. However, it is unclear if water containing Mn was used to reconstitute the formula which may have influenced levels of Mn in hair.

Findings supporting the exposure – outcome relationship between Mn concentrations in water, hair and child neurodevelopment were reported in three papers. Bouchard et al. reported higher levels of hair-Mn in children whose well water had higher Mn levels [32], and higher levels of Mn in water and hair were significantly associated with lower IQ scores. A 10-fold increase of Mn intake from water consumption was associated with a decrease of 2.5 IQ points (95 % CI:-3.9, -0.9; *p* < 0.01) among 9 year olds [32]. Oulhote et al. reported higher concentrations of Mn in hair and water were associated with poorer scores on memory, attention and motor function from the same population [47]. Average Mn water levels in this study were lower than the earlier study (20 micrograms/L. vs 300 micrograms/L.). do Nascimento et al. also reported higher levels of Mn in hair and household tap water were associated with poorer IQ scores in children 6–12 years [37].

Only one study reported blood levels of Mn associated with both a measured environmental source and neurodevelopmental outcomes. Lucchini et al. reported levels of Mn in blood and hair were both positively associated with tremor intensity in the dominant hand; the authors also found a borderline association between soil-Mn and tremor intensity [33]. Comparisons between the exposed and reference communities revealed average concentrations of Mn in soil (958 ppm versus 427 ppm), respectively. The authors describe metals in soil as good indicators of general environmental insult given their stability over time in the environment reflecting both background soil deposition and cumulative inputs from atmospheric deposition of historical industrial emissions.

Two additional studies reported higher levels of Mn in both the hair and the blood of children who lived near an industrial source of Mn [15, 31]. Haynes et al. reported low and high Mn levels in blood and hair were associated with lower full IQ and subscale scores, with significant negative associations between the highest versus middle two quartiles of blood-Mn (ß -3.51; 95 % CI: -6.64, -0.38) and hair-Mn (ß -3.66; 95 % CI: -6.9, -0.43) and full scale IQ in children ages 7–8 years [15]. Riojas-Rodriguez et al. found hair-Mn was inversely associated with verbal IQ (ß -0.29; 95%CI: -0.51, -0.08), performance IQ (ß -0.08; 95%CI: -0.32, -0.16), and total IQ (ß -0.20; 95%CI: -0.42, 0.02), in children ages 7–11 years [31]. The authors reported the 24 h median Mn in PM10 in exposed communities (0.13 micrograms/m^3^) was higher than the exposed communities (0.02 micrograms/m^3^).

Finally, Kahn et al. reported an inverse association between Mn in drinking water and children’s annual test scores in mathematics [58]. Levels of Mn in water above 400 micrograms/L (the former WHO standard) was associated with a 6.4 percentage score loss (95 % CI = 0.5, 12.3) in test scores. This study did not test any Mn biomarkers, but a prior paper showed a lack of association between blood-Mn and water-Mn [29].

## Discussion

A growing body of literature has examined the association of increased levels of Mn with neurodevelopmental effects in children from across the world. The evidence is most consistent in studies reporting decrements in IQ scores among primary school-aged children exposed to excessive levels of Mn. However, the inconsistency of findings in other studies reflects, in part, the considerable variation in study design including the source of Mn (water, air, or soil), exposure pathway (ingestion or inhalation), biomarkers measured (blood, hair, teeth, urine), study population (age, sex, and developmental and nutritional status) and neurological outcomes examined (IQ, motor skills, infant or early childhood development).

A recent pilot study tested the use of fMRI to reveal specific brain changes associated with Mn exposure. The findings revealed long-term exposure to Mn in the first stage of life can decrease olfactory function. There was also evidence that Mn exposure can adversely affect the functionality of the limbic system which the authors describe as suggestive of an alteration of the brain network in addressing emotional responses [59]. While scientifically promising, this approach may be less feasible for large, population studies of infants and young children given the expense and potential resistance of parents to having their children scanned for research in the absence of disease. However, with further testing in larger samples this approach could complement the use of biomarkers in studies of Mn exposure.

While relatively few studies investigated Mn exposures with biomarkers and neurodevelopment outcomes in infants, those studies using prospective study designs provided compelling evidence of the adverse effect of Mn. Biomakers of Mn using cord blood or serum provided a temporal association between fetal Mn exposures and later outcomes including cognitive and language development scores in 2 year olds [28], attention and nonverbal memory and hand skills in 3 year olds [43], and behavioral neurological development in newborns [57].

Measurement of Mn deposits in shed teeth provided insights more precise than those of cord blood or serum into the timing of early life exposures. While the CHAMACOS study is a large and comprehensive study of potential neurodevelopmental effects from pre-and postnatal dentin-Mn exposure in school-aged children [39, 45], the findings raise questions as the direction of the effects observed with higher levels pre- and postnatal Mn included both adverse effects with behavioral outcomes and positive effects with better memory abilities [39] inconsistent with other studies of school age children reporting higher Mn levels associated with poorer memory [4, 16, 54] and cognitive outcomes [15, 16, 31, 32, 35–37]. These authors posit the inconsistent findings may be due to differences in the exposure matrix used to quantify Mn levels or Mn exposure pathways or possibly that the levels of Mn in their sample could be within the range at which Mn acts as a beneficial nutrient rather a than a neurotoxicant suggesting a need for additional research [39].

Based on the studies reviewed here, hair-Mn was the most frequently examined biomarker, and it was consistently associated with lower child IQ scores suggesting hair may be the most consistent and valid biomarker for Mn to date for children in population studies. While blood-Mn was associated with a range of neurodevelopmental outcomes, the findings across studies were inconsistent.

Bouchard and colleagues acknowledged the lack of consensus on an optimal biomarker of exposure to Mn and blood-Mn levels can vary widely in the short-term and likely does not reflect long-term exposure [32]. Oulhote et al. reported that blood and urine are poor measures of Mn exposure [47].

In contrast, hair-Mn is posited by these investigators as a more consistent and valid biomarker of Mn. Bouchard et al. reported that hair-Mn will reflect the metal uptake averaged over the duration of the follicle formation although the mechanism of Mn uptake into hair is not well understood [32]. Hair typically grows 1 cm per month thereby providing an exposure estimate of 1–6 months [15]. Lucchini et al.’s preliminary analysis of hair biomarkers of Mn suggests it may be a better measure of integrated exposure and body burden over the prolonged period of hair growth, relative to biomarkers of blood or urine Mn, due to its rapid homeostatic control [33]. However, variability in hair-Mn concentrations may be related to various factors including difference in exposure, pharmacokinetics, hair pigmentation and issues of sample collection and cleaning [15]. Hair analysis for Mn requires rigorous cleaning procedures to minimize contribution of external Mn contamination without comprising endogenously incorporated Mn [33, 60].

Interpretation of Mn levels in hair must be carefully evaluated because Mn levels may be higher in some hair types than others (i.e., in darker hair), and because dye, bleach or other topical treatment may either contaminate hair or effect Mn incorporation into its structure [61, 62], although topical hair treatment is less relevant for studies of children. Additionally, in a pilot study in progress we have found some infants lack sufficient hair to analyze.

The literature also lacks sufficient analyses of the connections between the environmental source, the internal dose and the associated neurodevelopmental and cognitive outcomes. Studies reported findings supporting the exposure-outcome relationship between Mn concentrations in water, hair and adverse outcomes in child neurodevelopment [32, 37, 47]. In contrast, investigators who collected data on well water-Mn, blood-Mn and neurological outcomes failed to demonstrate an association between Mn concentrations in water and blood [18, 29, 30]. However, a statistically significant and dose-dependent association between water-Mn concentrations and IQ scores (Full Scale, Performance and Verbal) was reported [18]. This result is important as it provides strong evidence that ingestion of drinking water is a major source of environmental Mn potentially related to adverse neurodevelopment.

Few studies provided evidence of the association of environmental sources, biomarkers of Mn and neurodevelopment outcomes. Torres-Agustín et al. reported significantly higher Mn in blood and hair in an exposed (versus control) group with respiratory exposure to fine particulate matter of 2.5 microns or less in width, although only hair-Mn was significantly associated with poorer neurological outcomes [54]. This is important, though they did not report how increases in air Mn content affected Mn biomarker levels. However, Lucchini et al. reported evidence of both blood-Mn and hair-Mn being associated with increased tremor intensity in the dominant hand, and a borderline association between soil-Mn and tremor intensity. These authors report that the soil-Mn reflects past or cumulative exposures [33]. Future studies also need to quantify the association between environmental sources and selected biomarkers.

Finally, it is important that the continuum of exposure is carefully measured given the possibility of an inverted U-shaped association between Mn exposure and children’s health, neurodevelopment and cognitive outcomes. Ultimately, if public health programs are to provide prevention guidance for specific exposure sources such as drinking water, PM10 and soil regarding over-exposure to Mn, the threshold of beneficial Mn exposure must also be identified to ensure children receive the optimal benefit and the safe limit relative to their age and duration of exposure.

## Conclusion

With evidence mounting for the negative impact of Mn on children, research is needed to address the gaps in the literature that would help elucidate safe levels of Mn exposure for fetuses, infants and children. There is a particular need for a consistent measurement approach to biomarkers of Mn, as well as for environmental exposure sources and neurological outcomes, to make research findings comparable across studies. Additionally, feasibility issues are important when selecting biomarkers of exposure. The most promising Mn biomarker to date for the study of children is hair, but hair collection is not feasible for all infants and cleaning exogenous contamination of hair requires particular attention to evidence-based procedures. While cord blood appears an effective biomarker for measuring fetal exposure, it is logistically challenging and expensive to collect if study participants give birth at multiple hospitals. The use of teeth as a biomarker of Mn is intriguing, but it requires a minimum of 8 years from enrollment of pregnant women before children start to shed teeth that can be analyzed for Mn concentrations. The scientific and practical challenges of selecting the best biomarkers of Mn in children suggests the need for novel applications of additional biomarkers of chronic exposure to Mn. to help inform the science and ultimately determine public health prevention policies particularly for fetuses and infants given their heightened vulnerability to excessive Mn.

## Abbreviations

ADHD: Attention deficit hyperactivity disorder
As: Arsenic
B-Mn: Blood biomarker of manganese
B-Pb: Blood biomarker of lead
CBC: Children’s behavioral checklist
Cd: Cadmium
CHAMCOS: Center for Health Assessment of Mothers and Children of Salinas
CI: Confidence interval
cm: Centimeter
Cu: Copper
Fe: Iron
FeS: Iron sulfide
g: Gram
GM: Geometric mean
H.A.: Health advisory
Hair-Fe: Hair biomarker of iron
Hair-Mn: Hair biomarker of manganese
Hair-Pb: Hair biomarker of lead
HD: Hyperkinetic disorder
Hg: Mercury
I.Q.: Intelligence quotient
L: Liter
m3: Cubic meter
MDI: Mental Development Index, Bayley Scales of Infant Development
Mg: Magnesium
mg: Milligram
Mn: Manganese
NS: A statistically nonsignificant association
Pb: Lead
PDI: Psychomotor Development Index, Bayley Scales of Infant Development
PM10: Particulate matter with a diameter of 10 micrometers or less
PM2.5: Particulate matter less than 2.5 microns in width
SD: Standard deviation
SES: Socioeconomic status
WASI: Wechsler Abbreviated Intelligence Test
Water-Mn: Water as an environmental measure of manganese
WISC: Wechsler Intelligence Scale for Children
Zn: Zinc
